# The miRNA Mirage: How Close Are We to Finding a Non-Invasive Diagnostic Biomarker in Endometriosis? A Systematic Review

**DOI:** 10.3390/ijms19020599

**Published:** 2018-02-17

**Authors:** Swati Agrawal, Thomas T. Tapmeier, Nilufer Rahmioglu, Shona Kirtley, Krina T. Zondervan, Christian M. Becker

**Affiliations:** 1 Endometriosis CaRe Centre, Nuffield Department of Women’s and Reproductive Health, University of Oxford, Oxford OX1 2JD, UK; drswatiagrawal2010@gmail.com (S.A.); thomas.tapmeier@obs-gyn.ox.ac.uk (T.T.T.); krinaz@well.ox.ac.uk (K.T.Z.); 2 Wellcome Trust Centre for Human Genetics, University of Oxford, Oxford OX1 2JD, UK; nilufer@well.ox.ac.uk; 3 Centre for Statistics in Medicine, Nuffield Department of Orthopaedics, Rheumatology, and Musculoskeletal Sciences, University of Oxford, Oxford OX1 2JD, UK; shona.kirtley@csm.ox.ac.uk

**Keywords:** endometriosis, microRNA, circulating, non-invasive, biomarker

## Abstract

Background: Endometriosis is a common disorder of the reproductive age group, characterised by the presence of ectopic endometrial tissue. The disease not only causes enormous suffering to the affected women, but also brings a tremendous medical and economic burden to bear on society. There is a long lag phase between the onset and diagnosis of the disease, mainly due to its non-specific symptoms and the lack of a non-invasive test. Endometriosis can only be diagnosed invasively by laparoscopy. A specific, non-invasive test to diagnose endometriosis is an unmet clinical need. The recent discovery of microRNAs (miRNAs) as modulators of gene expression, and their stability and specificity, make them an attractive candidate biomarker. Various studies on miRNAs in endometriosis have identified their cardinal role in the pathogenesis of the disease, and have proposed them as potential biomarkers in endometriosis. Rationale/Objectives: The aims of this review were to study the role of circulatory miRNAs in endometriosis, and bring to light whether circulatory miRNAs could be potential non-invasive biomarkers to diagnose the disease. Search methods: Three databases, PubMed, EMBASE, and BIOSIS were searched, using a combination of Mesh or Emtree headings and free-text terms, to identify literature relating to circulating miRNAs in endometriosis published from 1996 to 31 December 2017. Only peer-reviewed, full-text original research articles in English were included in the current review. The studies meeting the inclusion criteria were critically assessed and checked using the QUADAS-2 (Quality Assessment of Diagnostic Accuracy Studies) tool. The dysregulated miRNAs were assessed regarding the concordance between the various studies and their role in the disease. Outcomes: Nine studies were critically analysed, and 42 different miRNAs were found to be dysregulated in them, with only one common miRNA (miR-20a) differentially expressed in more than one study. miR-17-5p/20a, miR-200, miR-199a, miR-143, and miR-145 were explored for their pivotal role in the aetiopathogenesis of endometriosis. Wider implications: It is emerging that miRNAs play a central role in the pathogenesis of endometriosis and have the potential of being promising biomarkers. Circulating miRNAs as a non-invasive diagnostic tool may shorten the delay in the diagnosis of the disease, thus alleviating the suffering of women and reducing the burden on health care systems. However, despite numerous studies on circulating miRNAs in endometriosis, no single miRNA or any panel of them seems to meet the criteria of a diagnostic biomarker. The disagreement between the various studies upholds the demand of larger, well-controlled systematic validation studies with uniformity in the research approaches and involving diverse populations.

## 1. Introduction

Endometriosis is an oestrogen dependent common inflammatory disorder characterised by the presence of endometrial glands and stroma outside the uterine cavity. Typical clinical symptoms include severe chronic pelvic pain, dysmenorrhoea, dyspareunia, and infertility [1]. It is estimated to affect up to 10% women of the reproductive age group, accounting for approximately 176 million women worldwide [2,3]. The prevalence rises to 20% in patients with chronic pelvic pain, and to 30–50% in patients undergoing fertility treatment [4,5,6]. No correlation exists between symptom intensity and the severity of endometriosis using the widely applied ASRM surgical staging system, and endometriosis can be asymptomatic, even in patients with severe forms of the disease [7]. The disease can cause enormous physical and mental suffering to the affected women and their partners, impairing the ability to work and increasing sick days, and therefore, leading to considerable burden to health care systems and society [8]. 

### 1.1. The Need for a Biomarker

Too little is known about the pathophysiology responsible for the establishment, development, and course of disease progression. In addition, due to the combination of the non-specific nature of endometriosis-associated symptoms, insufficient public awareness, the intra-abdominal location of endometriotic lesions, and lack of clinically relevant biomarkers, the average lag phase between the onset of symptoms of endometriosis, and its diagnosis, has been estimated to be 8 years in the United Kingdom, and 9 to 12 years in the United States [9,10,11]. The delay in the diagnosis may lead to increased severity of the disease, although spontaneous regression has been described [12,13]. The diagnosis can only be established by direct visualisation of the lesions during laparoscopic surgery, ideally with histological confirmation [10]. Therefore, the discovery of a non-invasive biomarker or a panel of such would hasten the diagnosis of endometriosis, decrease the need for surgery, and as such, the risk of surgical morbidity, allow non-invasive monitoring, and may open ways for various therapeutics. In addition, they might have a purpose in evaluating the recurrence risk, thereby enabling secondary prevention strategies. Finally, early diagnosis may also influence the timely decision-making regarding fertility issues. Several potential biomarkers have been tested, but none of them has proven to be sufficiently sensitive and specific for clinical use [14]. To date, discovering a non-invasive diagnostic test with requisite specificity remains the major priority of research in endometriosis [15].

### 1.2. Endometriosis: A Systemic Disease

Emerging evidence indicates that endometriosis is a systemic disease with widespread effects beyond the usual gynaecological manifestations, which might represent just the tip of the iceberg [16]. Women with endometriosis have a complete range of diseases with underlying immune and hormonal basis. They have a heightened risk of certain disorders, such as fibromyalgia, chronic fatigue syndrome, allergies [17], infections, carcinoma of ovary and breast, non-Hodgkin lymphoma, and melanoma [18,19,20,21,22]. The disease is associated with altered humoral as well as cell-mediated immunity, as revealed by the increase in immune cells, overproduction of various pro-inflammatory mediators, cytokines and growth factors, and the presence of non-specific immunoglobulins in peritoneal fluid and circulation [23,24,25]. These inflammatory changes are systemic, and not merely confined to the peritoneum [26]. These aspects indicate the key role of chronic systemic inflammation in the pathogenesis of the disease process [27]. It is postulated that the underlying immune disorders may affect the eutopic endometrium, as well as favour the growth of ectopic endometrial lesions. Moreover, the dysfunctional immune system in the affected women is responsible for the higher incidence of auto-immune disorders [28], like Hashimoto’s thyroiditis, Grave’s disease [29], systemic lupus erythematosus, rheumatoid arthritis [30], and multiple sclerosis [17]. Recently, many studies have reported significant dysregulation of microRNAs in the circulation in women with endometriosis [31,32].

### 1.3. MicroRNAs (miRNAs)

**Figure 1:**
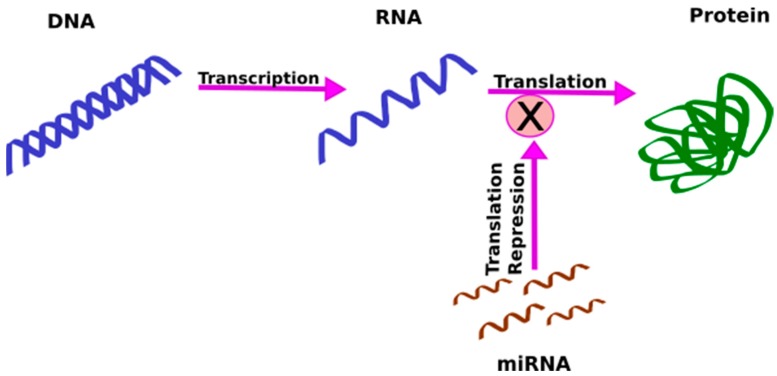
miRNAs regulate gene expression. They inhibit translation of the target messenger RNA (mRNA), and thus, repress protein synthesis.

Increasing evidence suggests that genetic factors play a significant role in the pathophysiology of endometriosis; however, the implicated genetic loci typically reside in intergenic or intronic regions that may regulate gene expression [33,34], rather than in coding regions. An important class of molecules that are involved in the regulation of gene expression are miRNAs. miRNAs are single-stranded, highly conserved, non-coding RNAs, approximately 21–25 nucleotides long, that bind to their complementary messenger RNA (mRNA), and through regulation of mRNA degradation, repress translation [35] (Figure 1). Secreted within extracellular vesicles by cells, they can act as cell-to-cell messengers and carry information between cells.

The seminal discovery of miRNAs in 1993 by two independent researchers, Lee et al. and Wightman et al., in *Caenorhabditis elegans*, was a breakthrough in the field of genetics and biomarkers [36,37]. Since then, 28645 precursor and 35828 mature miRNAs from 223 species have been described and included in the latest miRBase 21 [38]. miRBase is a comprehensive biological database archiving all the published miRNA sequences and annotations [39,40,41]. The latest version, miRBase 21, launched in June 2014, and holds 2603 mature human miRNAs; however, only 541 have been labelled as high confidence miRNAs, while the rest need to be validated further [38]. Mechanistically, miRNAs bind to the 3′ untranslated region (3′ UTR) of the target mRNA, and recognise the mRNA with the help of nucleotides from position 2 to 7 called “RNA seed” [42]. More than 60% of human protein coding genes have conserved sites for miRNA binding and, considering some non-conserved sites, too, most of the human genes are probably regulated by miRNAs [43]. The biogenesis of miRNAs is tightly regulated, and any aberration can cause various diseases, including cancer. 

So far, approximately 2000 miRNAs are known to influence the pathogenesis of various diseases in humans. Their proportion and amount largely determine the degree of expression of the target mRNA. miRNAs are of intracellular origin, and they play a vital role in various physiological and pathological processes, including cell-to-cell signalling, cell division, differentiation, and death [44,45,46,47]. They are released from cells into the circulation [48], chaperoned by carriers like Argonaute, nucleophosmin 1 (NPM1), and high-density lipoproteins, or packed into extracellular vesicles, e.g., exosomes [49,50]. This binding of circulating miRNAs to carriers prevents their degradation by ribonucleases (RNases) present in blood and other biofluids. The circulating miRNAs are taken up by recipient cells, where they repress translation. Evidence suggests that a miRNA might enhance the translation of the target mRNA, though it is quite infrequent [51]. 

### 1.4. Biogenesis of miRNAs

**Figure 2:**
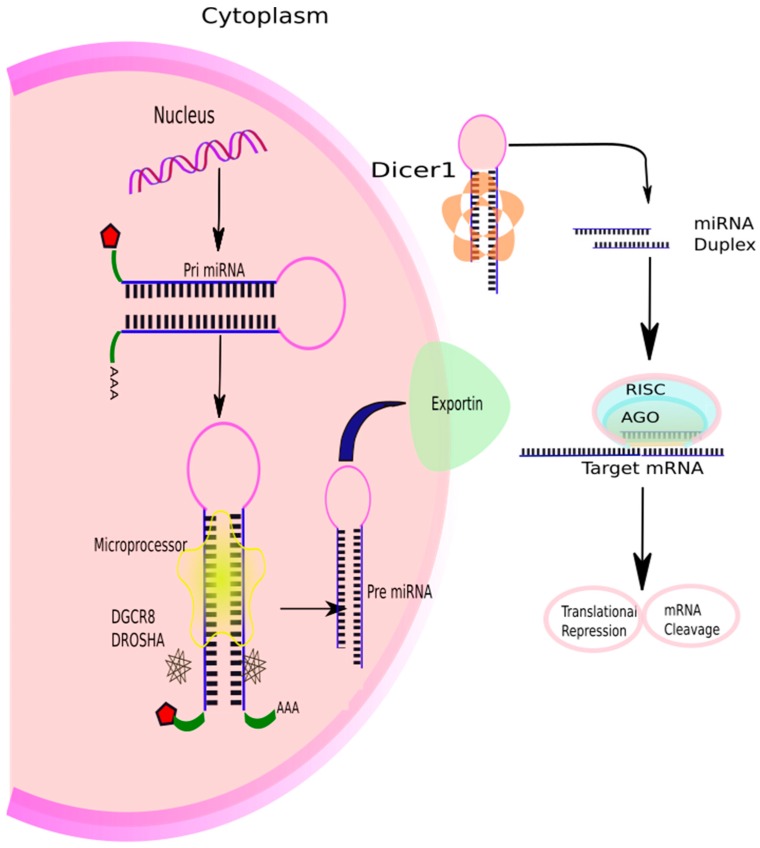
Overview of microRNA (miRNA) biogenesis and function. miRNAs are transcribed as primary miRNA (pri-miRNA) in the nucleus by the RNA polymerase II (Pol II) enzyme. The pri-miRNA is then cleaved by the microprocessor complex formed by an RNase III enzyme, Drosha, and RNA binding cofactor, Pasha, to form precursor miRNA (pre-miRNA). Pri-miRNA is exported to the cytoplasm by exportin 5, where the miRNA duplex is cleaved by Dicer, and then unwound by helicase to form a 19–22 nucleotides long mature miRNA. The guide strand gets incorporated into RNA-induced silencing complex (RISC), and the complex regulates post-translational modification by binding to the target miRNA.

The precise regulation of gene expression via miRNAs has been a topic of intense research. Acting via finely regulated complex network, an individual miRNA can regulate more than a hundred mRNAs [52], and each mRNA can be the target of numerous miRNAs [53], thus, a few hundred genes are being regulated by each miRNA [42]. The biogenesis of miRNAs is very intricately regulated at various checkpoints, and is possibly the cardinal regulatory mechanism underpinning gene expression. Usually, miRNAs are transcribed from genes in the intronic region of coding or non-coding transcripts, but some of them are also coded from exons [54]. In some cases, the miRNA gene is transcribed together with the host gene, thus providing coupled regulation along with the protein coding gene [54,55]. The majority of miRNAs are transcribed by RNA polymerase II (Pol II) to form several hundred nucleotides-long primary miRNAs (pri-miRNA) [56] which are capped at the 5′ end, polyadenylated, and spliced [57] (Figure 2). A pri-miRNA can generate up to six miRNAs. 

They fold into long hairpin looped structures, which are recognised by the RNA-binding protein co-factor, DiGeorge syndrome critical region 8 (DGCR-8), or Pasha. Pasha gets incorporated with double-stranded (ds) RNase III enzyme, Drosha, to form a microprocessor complex [58,59,60]. Drosha cleaves dsRNA towards the base of stem-loop in the pri-miRNA to release an approximately 70 nucleotides long hairpin-shaped precursor miRNA (pre-miRNA), which has two nucleotides overhanging on the 3′ end of the miRNA. Pre-miRNAs are transported from the nucleus to the cytoplasm by a shuttler, exportin-5, in an energy-dependent process [61,62]. Exportin-5 recognises the two-nucleotide overhang in the pre-miRNA. In the cytoplasm, an RNase III enzyme, Dicer, cuts away the loop at the 3′ and 5′ ends, leaving an asymmetrical miRNA duplex, about 22 nucleotides in length, with 3′ overhangs of two nucleotides [63,64,65]. The duplex is then unwound by helicase enzyme to form two miRNAs, one of which usually gets degraded. Only one of the two strands of a mature miRNA binds to the Argonaute protein and gets incorporated into the RNA-induced silencing complex (RISC) [66]. miRNA guides the complex to bind to target mRNA, and the genes are silenced either by translational repression or mRNA cleavage [66,67]. The degraded mRNAs are stored in small cytoplasmic structures called P-bodies for degradation [68]. 

### 1.5. miRNAs as Candidate Biomarkers 

Several studies have found miRNA signatures representative of diseases in various diseased tissues, as well as urine, serum, plasma, and other body fluids in numerous pathologies, making them an appropriate candidate for biomarkers [69,70,71]. As many miRNAs are tissue specific, their systemic dysregulation in peripheral blood points towards a distinct pathology [72,73,74]. miRNAs are now emanating as leading biomarkers, not only for diagnostic purposes, but also for disease stratification and therapeutics. It has also been observed that detecting a small number of miRNAs gives more information about the disease than studying the expression of several mRNAs.

An ideal biomarker is one which is specific to the disorder, can be detected early in the disease process, accessible from peripheral tissue (non-invasive), stable, reproducible, and associated with a known mechanism [75]. There are many challenges to identify new protein-based biomarkers, due to the complexity of the structure of a protein and various post-translational modifications. miRNAs may be more attractive as biomarkers, due to their lower complexity, tissue specificity, and no known post-translational modifications [76]. They are stable in blood, urine, and tissues, and can therefore serve as possible biomarkers for many conditions, including endometriosis. However, the precision and accuracy of miRNA measurement are not without challenges. A miRNA is a very short sequence of nucleotides with highly variable GC content, which leads to different hybridisation properties and makes its detection very demanding [77]. Furthermore, their minuscule amount present in serum or urine requires further advancements in technology to develop robust, precise, and highly sensitive techniques for miRNA detection. Despite the limitations, the discovery of miRNAs has opened new horizons in the unravelling the pathomechanisms of various diseases, and has given a whole new dimension to the field of biomarkers.

### 1.6. Various Techniques of miRNA Profiling

The relative expression of miRNAs can be studied by various techniques. Usually, miRNA profiling involves three major phases: discovery phase, validation phase, and functional analysis. In the discovery phase, miRNA expression profile is screened for differentially expressed miRNAs between cases and controls, to allow for maximum possible targets. This is followed by a validation step, in which the findings of the first step are corroborated employing more sensitive techniques. Finally, the functional relevance of miRNAs and the mRNA–miRNA relationship is explored utilising in silico analysis. Usually, hybridisation-based methods, like microarrays, are used as an initial approach to finding the potential candidate miRNAs; real-time PCR (qRT-PCR) is often used subsequently to validate highly dysregulated miRNAs among the distinct profile obtained using microarrays. qRT-PCR, due to its high precision, accuracy, and vast range, remains the gold standard for miRNA quantification [78,79]. miRNAs can be sequenced using next-generation sequencing (NGS) platforms, in which after reverse transcription, millions of DNA fragments are sequenced in parallel. A range of platforms can be used for miRNA sequencing, including SOLiD (Applied Biosystems), Solexa, HiSeq, MiSeq, MiniSeq, NextSeq (Illumina), and Ion Torrent (Invitrogen), to name a few. Using bioinformatics, these fragments are aligned and mapped, and their expression levels are analysed, thus eliminating the need for sequence specific hybridisation probes which are required in a microarray. Moreover, NGS has the advantages of high sensitivity and resolution, and excellent reproducibility [80], though considerable computational support is required. The biggest drawback is that there is a great variation in the performance among the different platforms. Mestdagh et al. (2014) have shown that hybridisation-based platforms exhibit lower sensitivity, irrespective of input RNA amount, while sequencing-based platforms display high sensitivity in the presence of ample of RNA, and the sensitivity is lost, if the amount of RNA is limiting. Thus, there are considerable interplatform differences, with different pros and cons for each technique [81].

### 1.7. miRNA Profiling Studies in Endometriosis

Recent evidence suggests that dysregulation of microRNA (miRNA) may be central to disease pathogenesis in endometriosis [82]. miRNAs are likely to regulate mRNAs and molecular networks that contribute to the key pathways proposed in the pathophysiology of endometriosis, like inflammation, angiogenesis, tissue repair, and extracellular matrix [82]. Moreover, one miRNA can target many mRNAs, altering several aspects of a disease process [83]. Women with endometriosis have distinct circulating miRNA signatures, as identified in numerous studies [84]. However, the source of these circulating miRNAs and their role in disease pathogenesis is yet unclear. Evidence suggests that they may be released from damaged cells after cell death or disruption of cellular integrity [85]. Other studies propose that miRNAs may be actively secreted from cells and play a vital role in intercellular communication [86,87]. Similar to hormones and proteins, and in fact, many molecules, miRNA profiles in endometrium are likely to alter with the different phases of the menstrual cycle [88,89,90]. In addition, differences in the eutopic endometrial miRNA profile between women with and without endometriosis have been explored [91,92,93,94]. Many studies have investigated the role of circulating miRNAs in endometriosis, but the results on dysregulated miRNAs have been inconsistent, and thus none of the findings have been translated into clinical practice. 

Here, we present a systematic review on circulating miRNAs in endometriosis. The aims of this review are to study the role of circulatory miRNAs in endometriosis; to determine miRNAs with pivotal role in the disease; to address their functional relevance by delineating the miRNA–mRNA relationships; to establish the concordance between the various studies; to explore the relation of the abundance of the miRNAs between tissue and circulation, and bring to light whether circulatory miRNAs could be potential non-invasive biomarkers to diagnose endometriosis. 

## 2. Results

A total of nine peer-reviewed studies were included and comprehensively reviewed in this analysis. All of them were from a single institution, except one, which was multi-institutional [95]. The study characteristics, like the country, mean age of the participants, study cohort, sample type, and stage of endometriosis, are described in Table 1.

The nine studies included analysed 357 patients with endometriosis and 255 women without the disease in total. The controls were from patients undergoing laparoscopy for various gynaecological indications, and not found to have endometriosis on laparoscopy. After evaluating each study, using the QUADAS-2 (Quality Assessment of Diagnostic Accuracy Studies) tool, the score ranged from moderate to high for all the included studies. Four were from China, three from the United States, one from Estonia, and one from Iran. The sample type was serum in five studies, and plasma in the remaining four. Three articles included patients from stage III–IV endometriosis; one considered mild to moderate endometriosis (stage I–II), while the rest included patients from all stages. One of the articles did not mention the stage of the disease at all [31]. The baseline characteristics of all the studies are listed in Table 2. Total RNA was extracted in all the manuscripts using various kits as described in Table 3. All except two used global miRNA profiling by microarray, followed by validation by qRT-PCR. The remaining two research studies analysed the expression of several chosen miRNAs using qPCR [95,96]. Different normalisation controls for qRT-PCR were utilised in the various studies, most common was U6snRNA [95,97,98]. Three of the nine studies found no significant difference in miRNA expression in different phases of the menstrual cycle [96,97,98]. Five of them did not study the cyclical changes in miRNA expression [31,32,99,100]. On the other hand, Cho et al. found significantly higher levels of let-7a, let-7d, let-7e, and let-7f in the secretory phase as compared to the proliferative phase in serum of women with endometriosis, while no such cyclic difference was observed in the control group. On comparing miRNA expression in endometriosis and control groups, in relation to menstrual cycle, the expression of let-7b, let-7c, let-7d, and let-7e was significantly lower in women with endometriosis than controls in proliferative phase. The techniques of miRNA profiling, internal controls used, and dysregulated miRNAs identified are detailed in Table 2. 

Overall, 21 different miRNAs were shown to be upregulated, and a similar number (21) downregulated in women with endometriosis, as compared to controls in the various studies. We found only a single circulating miRNA dysregulated in a similar direction, across more than one study, miR-20a (Table 3). The diagnostic data about the different miRNAs like sensitivity, specificity, and ROC AUC (receiver operating characteristic area under curve), if provided in the study, are tabulated in Table 4. The various dysregulated miRNAs could not be statistically evaluated, due to the lack of consensus among the published literature and the absence of statistical data presented in the manuscripts.

## 3. Discussion

miRNAs have emanated as the master regulators of various cellular processes, with a strong potential to be biomarkers with diagnostic and prognostic importance for many diseases. Several studies have investigated the role of miRNAs in endometriosis and their functional relevance, as revealed by in silico analyses. However, a mismatch between transcriptomes and proteins have been reported, and these corroborations are basically indirect [103]. This review aimed at exploring the potential role of circulating microRNAs (miRNAs) as non-invasive biomarkers in diagnosing endometriosis, and finding the candidate miRNAs with the highest potential to diagnose endometriosis non-invasively, which could further be validated in larger studies in light of the current evidence. The studies included in this report also give an insight into the relation between circulating and tissue miRNAs.

### 3.1. miRNAs with Differential Expression in Endometriosis

Most of the studies investigating circulatory miRNAs in endometriosis have initially profiled miRNAs by microarray, and then validated the most differentially expressed miRNAs by qRT-PCR. To minimise the false-positive rates, we have only analysed the unique miRNAs, which have been validated independently by qRT-PCR. Surprisingly, we found only a single miRNA (miR-20a), which was similarly dysregulated in two of the studies. There was no other miRNA with consistent direction of dysregulation in more than one study. miR-20a was found to be downregulated in plasma and serum of patients with endometriosis in two different studies [32,99]. Jia et al. (2013) reported 4.53-fold decrease in the level of miR-20a (*p*-value = 0.037). The area under curve (AUC) was 0.79, and at the cut-off value of 0.6879, the sensitivity and specificity were 60% and 90%, respectively. Whereas, Wang et al. (2016) recorded 1.87 FC (fold change) with a *p*-value of 3.81 × 10^−19^. A cut-off value was not mentioned, and the AUC was not calculated in the study. IsomiRs (arising from the two arms of the same precursor miRNA) of miR-141 (miR-141-3p and miR-141-5p) were found to be dysregulated in blood and serum respectively [96,97]. 

Additionally, two miRNAs (miR-15b-5p and miR-199a) were dysregulated in two different studies, but the direction was not concordant. miR-15b-5p was upregulated in plasma in the research by Suryawanshi et al. (2013), while it was downregulated in serum in an article by Wang et al. (2016). Similarly, two other studies reported differential dysregulation of miR-199a in serum of patients with endometriosis [97,100]. The discrepancy could be due to various reasons, including differences in study design, patient population, sample type, control group, stage of endometriosis, endogenous qRT-PCR controls, and platforms used for miRNA profiling. It has been shown that miRNA levels are higher in serum as compared to the corresponding plasma sample [104,105]. On the contrary, few studies reported higher concentration in plasma than serum [106,107,108], while many others found a strong correlation between plasma and serum miRNAs [109,110], and one study demonstrated no association between the two [111]. Additionally, miRNA levels in blood are affected due to haemolysis of the circulating cells [112], and since mild haemolysis is frequently seen, it is a primary source of error while measuring miRNAs in blood. Even the timing of sample collection seems to affect the circulating miRNA expression [96]. The authors observed lower plasma levels of miRNA-200a and miR-141, when the blood was collected in morning as compared to the evening samples. Finally, isomiRs (arising from the two arms of the same precursor miRNA) of miR-141 (miR-141-3p and miR-141-5p) were found to be dysregulated in blood and serum respectively [96,97]. 

### 3.2. Factors Influencing miRNA Expression

The translational regulation by miRNAs involves intricately regulated composite interactions in which a single miRNA regulates the transcription of many mRNAs, and a single mRNA can be influenced by multiple miRNAs [42]. The expression of miRNA in an individual is dynamic, and is influenced by an array of factors like age, ethnicity, physiological stage of body, presence of various diseases, smoking, and various other external factors [113,114,115,116,117,118]. Choosing an appropriate control group is crucial to study miRNA expression in circulation, and can prove very challenging. Endometriosis is a chronic inflammatory disease, and miRNAs specific for endometriosis as opposed to other inflammatory conditions arise from endometrial cells, and are released into the circulation bound to proteins or contained in extracellular vesicles, e.g., exosomes. Many miRNAs dysregulated in tissue and blood target mRNAs involved in inflammation. However, similar miRNAs (miR-9, miR-17-92 cluster, 125b, miR-199a-5p) have been proven to be significantly dysregulated in various other inflammatory and autoimmune disorders [119,120,121,122,123]. In order to minimise selection bias, all women with other inflammatory diseases, like PID (pelvic inflammatory disease) or any autoimmune disorders, should be excluded. Moreover, if healthy women are chosen as controls, they might harbour asymptomatic endometriosis, unless ruled out laparoscopically [124]. 

One of the important determinants of miRNA signature is phase of the menstrual cycle. Various studies in tissues have found significant differences in miRNA expression, and have related it to physiological processes, like cellular proliferation and endometrial receptivity [88,89,125]. Surprisingly, no such cyclic difference was observed in plasma of healthy women [126]. The authors speculated that the changes in miRNA expression at the cellular level in endometrium regulate the gene expression locally, and are insufficient to cause any detectable systemic changes. Analogously, no difference in miRNA signatures with menstrual cycle was seen in three of the nine studies [96,97,98]. Cho et al. observed significant cyclical variation in miRNA expression in women with endometriosis, although no difference was observed among the control group [95]. The same authors also found that let-7b, let-7c, let-7d, and let-7e were significantly downregulated in proliferative phase in women with endometriosis, as compared to the controls, while the level of miR-135a was lower in endometriosis group in the secretory phase.

Data normalisation is the backbone to obtain accurate results in qRT-PCR by minimising the effects of experimental variations [127,128,129,130]. The most commonly used manner is to use endogenous controls, which are invariably present in constant amounts across all samples as standards. The amount of a miRNA is measured, relative to an endogenous control, to eliminate the biological variations among the samples. Regrettably, there is no consensus regarding the choice of controls. U6snRNA is used by three of the studies in the review, but most of the others have used different controls, like miR-16, miR-132, 18s RNA, cel-miR-39, miR-30e, miR-99a, and miR-103-3p. Several studies noted significant fluctuations in the level of U6, and thus, recommended choosing other controls, like miR-16 and let-7a [131,132]. However, another study has found inconsistent levels of miR-16b, and suggested the use of RNU48, U75, and RNU44 as normalisation controls [133]. 

It is worthwhile to state that the majority of errors arise in the pre-analytical phase, and rigorous methodology needs to be implemented to minimise inaccuracies [134,135].

### 3.3. Candidate Circulating miRNAs in Endometriosis and Their Putative Role

A total of 40 miRNAs were found to be differentially regulated in the circulation in women with endometriosis in various studies. The targets of many of these miRNAs, and the underlying significance in the disease, are yet to be elucidated. The existing evidence on circulatory miRNAs with a role in the aetiopathogenesis of endometriosis is summarised. Their differential expression in endometrial tissues, as observed in the current literature, has also been described.

#### 3.3.1. miR-17-5p/20a 

**Figure 3:**
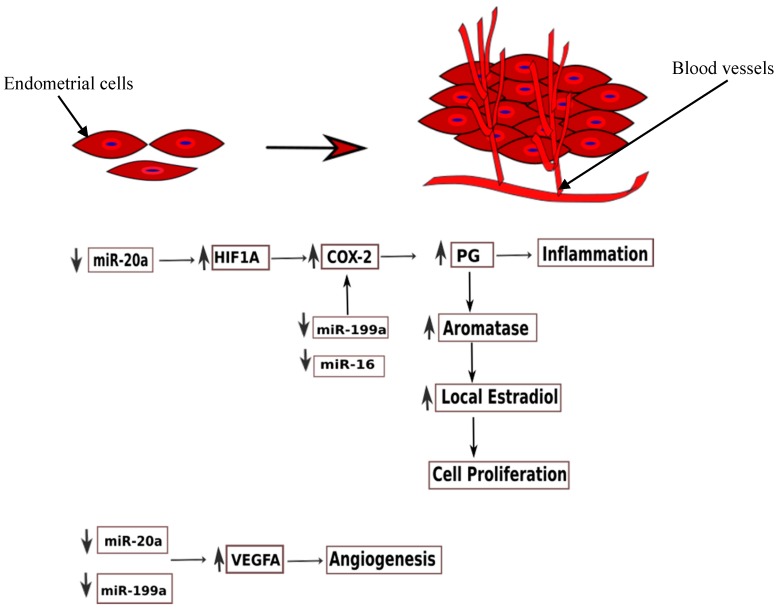
The figure schematically depicts the proposed role of miRNAs in angiogenesis and cell proliferation. ↓ indicates down-regulation and ↑ indicates up-regulation.

**Figure 4:**
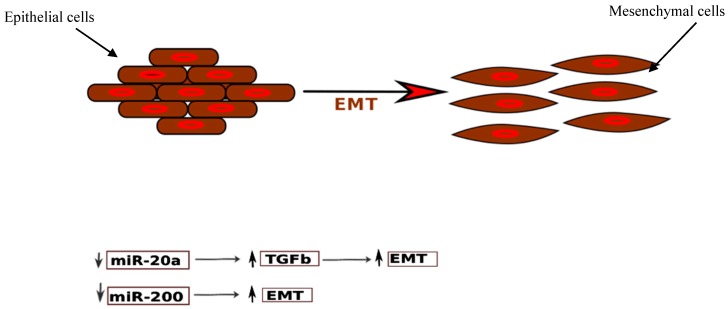
The figure schematically depicts the epithelial cells (endometrial cells) losing their polarity and cell to cell adhesion undergoing changes to assume a migratory mesenchymal cell phenotype. miR-20a and miR-200 play a crucial role in this epithelial mesenchymal transition (EMT) which also is proposed to be one of the key processes in the pathogenesis of endometriosis. ↓ indicates down-regulation and ↑ indicates up-regulation.

miR-20a was the only circulating miRNA with concordant downregulation in more than one study [32,99]. It is also shown to be downregulated in studies on endometrial tissues [84,136]. miR-20a is a part of the miR-17-92 cluster formed by six miRNAs (miR-17, miR-18a, miR-19a, miR-19b, miR-20a, miR-91a). miR-17-5p has been found downregulated in endometriotic tissue and plasma of women with endometriosis [99,136]. miR-20a, along with miR-17-5p, plays an important role in the aetiopathogenesis of endometriosis (Figure 3 and Figure 4). 

Hypoxia plays a crucial role in the development of endometriotic lesions [137]. Hypoxia stimulates the synthesis of prostaglandins, promotes angiogenesis, and modulates oestradiol signalling. Hypoxic stress induces the growth factors, like HIF (hypoxia inducible factor) and VEGF (vascular endothelial growth factor) [138]. These growth factors are the downstream targets of miR-17-5p/20a. HIF has two subunits (α and β) which form a heterodimeric complex. The decrease in the expression of miR-17-5p/20a eases the posttranscriptional suppression of its target mRNA, *HIF1A*, which further modulates the production of other proteins in cells under hypoxic stress. *HIF1A* stimulates COX-2 expression and prostaglandin production, thus promoting inflammation [137]. The other target of miR-17-5p/20a, VEGFA, promotes aberrant angiogenesis in endometriotic patches. This has been confirmed by demonstrating the constitutively elevated levels of HIF1α [139,140,141] and VEGF [142,143] in ectopic endometrial tissues. 

Another key feature of ectopic endometrial cells is augmented cell survival and reduced apoptosis. miR-20a negatively regulates the translation of BCL2 (B-cell lymphoma 2), which codes for an anti-apoptotic protein, and CDKN1A/p21 (cyclin-dependent kinase inhibitor 1A), a cell cycle repressor. In addition, miR-20a targets CCND1 (Cyclin D1) and E2F3 (E2F transcription factor 3), and thus participates in epithelial cell proliferation and decreased apoptosis. Furthermore, miR-20a targets TGF-β (transforming growth factor-β) and interleukin-8. Thus, downregulation of miR-20a leads to increased concentrations of these cytokines, which in turn, promote an inflammatory milieu and tissue repair, and thus contribute to the growth of endometriotic lesions. TGF-β also promotes epithelial–mesenchymal transition, which is a pathogenetic mechanism in endometriosis (Figure 4).

By contrast, miR-20a was found to be upregulated in endometriotic stromal cells [144]. miR-20a targets DUSP2 (dual-specificity phosphatase-2), and thus promotes phosphorylation of ERK. ERK, in turn, stimulates COX-2 expression, synthesis of prostaglandins (PGE_2_), and FGF-9 (fibroblast growth factor) expression. FGF-9 is a powerful mitogen, promoting the growth of endometrial and endothelial cells. This finally leads to cellular proliferation, angiogenesis, and development of endometriotic patches [144]. Additionally, PGE_2_ stimulates aromatase activity, and thus, the concentration of local oestradiol increases, in turn, causing cellular proliferation [145,146].miR-20a was also found upregulated in ovarian tissue of patients with ovarian endometriosis [147]. The authors proposed that miR-20a, along with miR-17-5p and other members of the cluster, downregulated TSP-1 (thrombospondin-1) and promoted neovascularisation. Another target mRNA of miR-20a is NTN4 (netrin-4), which has an antiangiogenic effect. Zhao et al. (2014) speculated that increased miR-20a reduces the expression of NTN4, and in turn, stimulates angiogenesis [147]. 

At this point, it is also noteworthy to mention that miR-17/20a is not specific for endometriosis, and its dysregulation has been reported in the context of various cancers like ovarian, cervical, hepatocellular, and colorectal carcinoma [148,149,150,151]. Therefore, a signature or a panel of miRNAs, rather than a single miRNA is capable of forming a robust biomarker to diagnose a disease with sufficient sensitivity and specificity, and to sufficiently demonstrate the intricacy of the disorder [152]. 

#### 3.3.2. miR-200 Family

The miR-200 family, consisting of miR-200a, miR-200b, and miR-141, have been found dysregulated in multiple studies in tissue, as well as the blood of patients with endometriosis. Rekker et al. (2015) reported significantly lower levels of all three miRNAs in plasma of patients with endometriosis [96]. The AUC for miR-200a, miR-200b, and miR-141 was 0.75, 0.67, and 0.71, respectively, with sensitivity of 71.9% for miR-141, and 90.6% for miR-200a and miR-200b [96]. The combined signature of all three miRNAs displayed sensitivity and specificity of 84.4% and 66.7%, respectively. Another study in tissue also found these miRNAs downregulated in ectopic endometrium as compared to the eutopic endometrium [84]. miR-200b exhibited decreased expression in endometrioma as compared to the eutopic endometrium [153]. 

**Figure 5:**
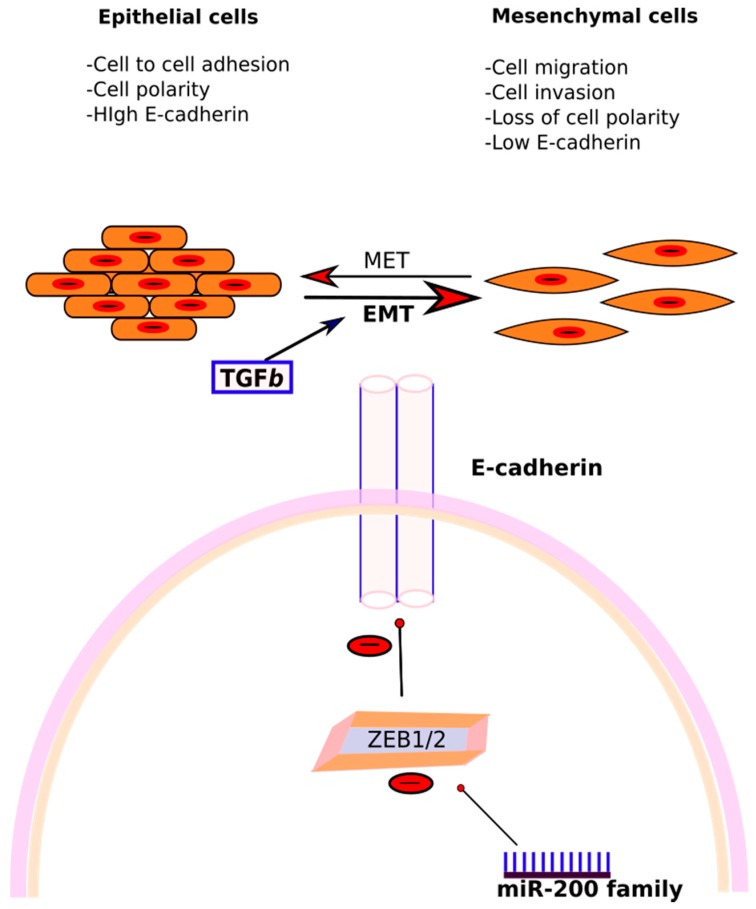
miR-200b regulates epithelial–mesenchymal transition (EMT). Downregulation of miR-200b in endometriosis upscales the translation of ZEB ½, which further inhibits E-cadherin expression on cells, and promotes EMT, contributing to the pathogenesis of endometriosis. TGF-β also promotes EMT and is found in higher levels in peritoneal endometriosis.

The role of the miR-200 family in endometriosis has been extensively studied. Ectopic endometrium possesses many characteristics of malignant cells, like invasiveness, high proliferation rate, and metastasis. In the embryo, endometrial cells are derived from intermediate mesoderm of the primitive germ cell layers after mesenchymal–epithelial transition (MET). According to the prevailing theory, the epithelial cells in pelvic endometriosis originate from retrograde menstruation of endometrial cells. Due to their origin, these are particularly prone to revert to their mesenchymal state. This epithelial–mesenchymal transition (EMT) is thus proposed to be one of the fundamental processes in initiating endometriosis [154]. In EMT, epithelial cells undergo a transition to a mesenchymal phenotype, and are then capable of breaching the basement membrane and reaching distant sites. The cardinal feature in EMT is the loss of proteins involved in adhesion, like E-cadherin (epithelial cell adherin) (Figure 5). Studies have found that cells in peritoneal endometriosis are E-cadherin negative, compared to the cells in the eutopic endometrium [155,156]. The expression of E-cadherin is decreased by various transcriptional repressors, like ZEB1 and ZEB2 (zinc finger E-box-binding homeobox 1 and 2), and SIP1 (Smad-interacting protein 1), which in turn, act as EMT activators. It has been found that the miR-200 family and ZEB1, ZEB2, and SIP1 reciprocally regulate each other in a negative feedback loop which governs EMT [157,158] (Figure 4). Thus, downregulation of miR-200b induces EMT by upregulating ZEB1 and ZEB2. In an experiment conducted on Madin Darby canine kidney (MDCK) epithelial cells, TGF-β1 treatment induced a morphological change with the loss of cohesion, decreased expression of E-cadherin, and elevated markers of mesenchymal origin, like fibronectin, ZEB1, and ZEB2 [158]. This was associated with a significant downregulation in the miR-200 family, pointing towards their role in EMT, and thus, potentially, endometriosis.

#### 3.3.3. miR-199a

The levels of circulating miR-199a-5p were significantly downregulated in a study by Hsu et al. (2014) (FC: 0.123; *p*-value: 0.025). In tissues, miR-199a was downregulated in ectopic endometrium and ovarian endometriomas, as compared to eutopic endometrium and controls, respectively [159]. miR-199a negatively regulates VEGF-A expression in tissues; thus, downregulation of miR-199a increases the translation of VEGF-A, and leads to its overexpression in tissues and blood. Dai et al. (2012) predicted the target of miR-199a-5p to be IκB kinase-β (IKBKB), which further activates nuclear factor-κB (NF-κB) and regulates the production of interleukin 8 (IL-8) [159]. Thus, downregulation of miR-199a would increase the expression of NF-κB and IL-8 production, and thereby enhance the invasive capability of endometrial cells. Additionally, decreased expression of 199a promotes COX-2 production and cell proliferation, as discussed previously. On the contrary, Wang et al. (2013) found higher levels of miR-199a in the serum of women with endometriosis (FC: 1.14 × 10^5^; *p*-value < 0.0001) [97]. The AUC for miR-199a was 0.825; sensitivity and specificity were 78.33% and 76.00%, respectively. They identified CLIC4, RTN4, and VCL as the target genes, and proposed that upregulation of miR-199a in endometriosis regulates adhesion and infiltration of endometrial cells by targeting these genes [97]. 

#### 3.3.4. miR-143 and 145

miR-143-3p and miR-145-5p form a cluster, and are upregulated in serum and tissue in various studies [82,98,160]. In the study by Cosar et al. (2016), the AUC for miR-143-3p was 0.926 with a *p*-value of <0.001 [98]. miR-143 targets FNDC3B (fibronectin type III domain containing 3B), which regulates cell motility [161]. An increased expression of miR-143 represses the transcription of FNDC3B, and thus, would promote cell invasion and migration in endometriosis [160]. In relation to miR-145, Cosar et al. found miR-145-5p to be upregulated, while Wang et al. found 145-3p to be downregulated in the patients with endometriosis. Another study found miR-145 to be downregulated in patients with stage 1 and 2 of endometriosis, while not in stage 3 or 4 [101]. 

### 3.4. Limitations of the Review

There are several limitations to this systematic review: not all the studies provided cut-off values, sensitivity, specificity, and AUC for the receiver operator characteristics of the various miRNAs. To minimise the false-positive rates, we have only analysed the miRNAs which are validated independently using qRT-PCR, which might cause missing of few relevant miRNAs. Also, we have also included studies which targeted specific miRNAs, rather than global profiling, which may introduce selection bias. There was no standardisation of the protocols or the techniques used among the various studies, which plays a pivotal role in analysing miRNA expression. 

### 3.5. Future Directions

Among the epigenetic players, miRNAs have emanated as powerful regulators of gene expression, and are investigated as diagnostic biomarkers for various diseases. This makes the systematic review timely. As discussed in the review, the minimal concordance rate among the studies could be due to the lack of standardisation in the study protocols, like sample collection, processing, various techniques used, and normalisation controls. The importance of standard operating procedures (SOPs) cannot be undermined. World Endometrioisis Research Foundation (WERF) has launched a global initiative Endometriosis Phenome and Biobanking Harmonisation Project (EPHect), to develop unanimity in sample collection and data recording in patients with endometriosis, which will facilitate comprehensive, global, internationally collaborative robust research in endometriosis [162,163,164,165]. Adhering to the SOPs would serve to minimise the pre-analytical errors. There is an absolute requirement of similar SOPs for studying RNA expression to develop unanimity among the studies. Only then would it be possible to establish a panel of miRNAs which could diagnose endometriosis non-invasively, with sufficient sensitivity and specificity. Pending these, circulating miRNAs could prove valuable not only in assessing prognosis and monitoring treatment, but also as major therapeutic targets in endometriosis. 

## 4. Materials and Methods

### 4.1. Search Strategy

Three databases, PubMed, EMBASE, and BIOSIS were searched to identify literature relating to circulating miRNAs in endometriosis. Search terms included variations of “endometrio”, “microRNAs”, “blood”, “serum”, “plasma”, “biomarkers”, “diagnosis”, and “prognosis” searched as Mesh or Emtree headings, where appropriate, or as free-text terms in the title, abstract, or topic fields. The search date range was 1996 to 31 December 2017, and no language restrictions were applied. The full search strategies for all databases are listed in Table S1.

**Figure 6:**
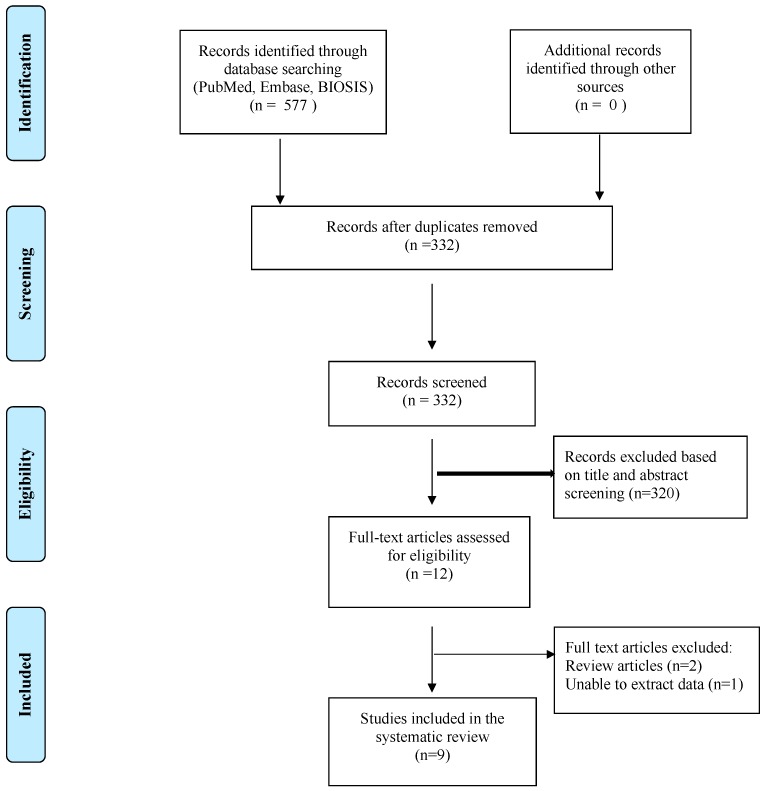
Search strategy and study selection as per PRISMA guidelines.

After careful evaluation, 568 articles were excluded. Eventually, only nine articles matched the previously described inclusion criteria and were included in the review (Figure 6).

### 4.2. Inclusion and Exclusion Criteria 

All studies were assessed for their relevance to endometriosis. Studies were included if they met all the following inclusion criteria: (1) original studies on humans available in English, published between January 1996 and December 2017; (2) assess the dysregulation of circulating miRNAs in endometriosis systematically and quantitatively; (3) analyse miRNA dysregulation in serum, plasma, or blood; (4) sample size in each study group is larger than 20 (Table 5). All articles on tissue miRNAs were excluded. Comparative studies, retrospective and prospective studies from peer-reviewed journals were included, whereas editorials, reviews, comments, letters, or unpublished studies were excluded. 

### 4.3. Quality Assessment 

The quality of all the included articles was assessed by QUADAS-2 (quality assessment for studies of diagnostic accuracy) and pre-specified inclusion and exclusion criteria. QUADAS-2 is an evidence-based bias assessment tool to evaluate the risk of bias and quality of diagnostic accuracy studies in a systematic review [166]. It incorporates four domains, including patient selection, index test, reference standard, and flow and timing. 

### 4.4. Data Extraction

All the study details, such as the name of the first author, place, duration of the study, year of publication, study design, sample type, sample size, method of miRNA extraction, qPCR technique, method of analysis, and normalisation control used in qRT-PCR were noted. Putative circulating miRNAs were determined; their cut-off values, *p*-values, fold change (FC), sensitivity and specificity of individual miRNAs were noted, if provided. miRNAs found to be dysregulated in more than two studies were interrogated regarding their role in endometriosis.

### 4.5. Ranking

All studies were analysed in terms of sample size, age of the participants, stage of disease, and research methodologies. All miRNAs that were found to be differentially regulated in endometriosis and validated using qRT-PCR were listed. A ranking method devised by Griffith et al. (2006) for genes was followed for miRNAs [167]: the highest preference was given to the miRNA consistently dysregulated in more than one study. The sample size of the study was next in the order of importance, followed by the magnitude of fold change of miRNA expression. 

### 4.6. Publication Bias

Publication bias is always a concern in systematic review, because all the research which takes place is not published. Studies with a significant result are more likely to be published compared to studies with null results. Moreover, well-controlled and properly carried out studies are less likely to achieve significance. In the current systematic review, only nine studies were included, and Begg and Egger funnel plots are unreliable in a review with less than ten studies [168]. 

The systematic review was registered with the International Prospective Register of Systematic Reviews (PROSPERO) at http://www.crd.york.ac.uk/prospero, registration number CRD42016048410. 

## 5. Conclusions

Based on the literature overview, circulating miRNAs seem to be promising candidates for a non-invasive biomarker for endometriosis. However, considerable discovery is yet to be done in this domain, and the techniques for miRNA profiling need to be further explored and standardised. The current disagreement between various studies as to methodology and results warrants the need for larger, well-controlled, systematic validation studies, with uniformity in research approaches, and involving a myriad of patient populations. The incongruity also accentuates the perplexity of this enigmatic disease, and highlights the necessity of novel techniques to design a reliable non-invasive test to diagnose endometriosis. We hope that our review might point towards the future oasis of true biomarkers, past the present miRNA mirage.

## Figures and Tables

**Table 1:** Characteristics of reviewed studies.

S. No	Author	Country	Sample Size	Sample Type	Mean Age (Years)		Stage of Endometriosis * in Cases	Characteristics of Controls
					Cases	Controls		
1	Suryavanshi et al. 2013 [31]	USA	Cases-33 Control-20	Plasma	NA	NA	NA	Healthy women
2	Jia et al. 2013 [99]	China	Cases-20 Controls-20	Plasma	34.1	32.1	III–IV	No evidence of endometriosis on laparoscopy
3	Wang et al. 2013 [97]	China	Cases-60 Controls-25	Serum	34.43	30.0	I–IV	No evidence of endometriosis on laparoscopy
4	Hsu et al. 2014 [100]	China	Cases-40 Controls-25	Serum	34.8	37.3	II–IV	No evidence of endometriosis on laparoscopy
5	Rekker et al. 2015 [96]	Estonia	Cases-61 Controls-65	Plasma	32.5	29.7	I–IV	35: No evidence of endometriosis on laparoscopy 30: Healthy
6	Cho et al. 2015 [95]	USA	Cases-24 Controls-24	Serum	33.08	32.16	III–IV	No evidence of endometriosis on laparoscopy
7	Cosar et al. 2016 [98]	USA and Korea	Cases-24 Controls-24	Serum	33.08	32.16	III–IV	No evidence of endometriosis on laparoscopy
8	Wang et al. 2016 [32]	China	Cases-30 Controls-20	Serum	34.0	32.5	I–II	No evidence of endometriosis on laparoscopy
9	Bashti et al. 2018 [101]	Iran	Cases-55 Controls-23	Plasma	28	28	I–IV	No evidence of endometriosis on laparoscopy

* Stage is according to the revised American Society of Reproductive Medicine classification of endometriosis [102]. NA: data not available.

**Table 2:** Research methodologies used in different studies.

Methodologic Parameter	Number of Studies (Out of 8 Manuscripts Analysed)
**Kits used for RNA extraction**	
mirVana	5
Trizol	1
miRNeasy	1
miRCURY	2
**Real-time PCR technique**	
SYBR Green	7
TaqMan	2
**Normalisation control in qPCR**	
Internal reference	8
External reference	1

**Table 3:** List of circulating miRNAs dysregulated in endometriosis after validation by qRT-PCR as found in various studies.

S. No	Author	Method	Normalisation Control Used in qRT-PCR	Dysregulated miRNAs
1	Suryavanshi et al. 2013 [31]	Global miRNA profiling using qRT-PCR followed by validation by qRT-PCR	miR-132	Up → miR-15b, 16, 191, 195, 362-5p, 1973, 1974, 1978, 1979, 4284
2	Jia et al. 2013 [99]	Microarray followed by qRT-PCR	miR-16	Down → miR-17-5p, 20a-5p, 22
3	Wang et al. 2013 [97]	Microarray followed by qRT-PCR	U6 snRNA	Up → miR-122, 199a Down → miR-9-3p, 141-5p, 145-3p, 542-3p
4	Hsu et al. 2014 [100]	Microarray followed by qRT-PCR	18s RNA	Down → miR-199a-5p
5	Rekker et al. 2015 [96]	qRT-PCR	miR-30e, 99a	Down → miR-141-3p, 200a-3p
6	Cho et al. 2015 [95]	qRT-PCR	U6 snRNA	Down → let-7b, miR-135a, let-7d, 7f
7	Cosar et al. 2016 [98]	Microarray followed by qRT-PCR	U6 snRNA	Up → miR-18a-5p, 125b-5p, 143-3p, 145-5p, 150-5p, 342-3p, 451a, 500a-3p Down → miR-3613-5p, 6755-3p
8	Wang et al. 2016 [32]	Solexa sequencing followed by qRT-PCR	cel-miR-39	Up → miR-185-5p, 424-3p Down → miR-15b-5p, 20a-5p, 30c-5p, 99b-5p, 127-3p
9	Bashti et al. 2018 [101]	qRT-PCR	miR-103-3p	Up→ miR-145 Down→ miR-31

Up—indicates upregulated miRNAs. Down—indicates downregulated miRNAs.

**Table 4:** Data on dysregulated miRNAs in various studies.

S. No	Author	miRNA	Cut-Off	AUC	Sensitivity (%)	Specificity (%)
1	Suryavanshi et al. 2013 [31]	miR-16 + miR-191 + miR-195	NA	0.90	88	60
2	Jia et al. 2013 [99]	miR-17-5p miR-20a miR-22 miR-17-5p + miR-20a + miR-22	0.9057 0.6879 0.5647 NA	0.74 0.79 0.85 0.90	60 60 90 NA	90 90 80 NA
3	Wang et al. 2013 [97]	miR-9-3p miR-122 miR-141-5p miR-145 miR-199a miR-542-3p miR-122 + miR-145 + miR-199a + miR-542-3p	NA NA NA NA NA NA NA	0.828 0.835 0.849 0.883 0.825 0.854 0.994	68.33 80 71.69 70 78.33 79.66 93.22	96 76 96 96 76 92 96
4	Hsu et al. 2014 [100]	ND	ND	ND	ND	ND
5	Rekker et al. 2015 [96]	miR-141 miR-200a miR-200b miR-141 + miR-200a + miR-200b	NA NA NA NA	0.71 0.75 0.67 0.76	71.9 90.6 90.6 84.4	70.8 62.5 70.8 66.7
6	Cho et al. 2015 [95]	let-7d	0.823	0.905	83.3	100
7	Cosar et al. 2016 [98]	miR-125b-5p miR-125b-5p + miR-451a + miR-3613-5p	0.0688	0.974 1	100 100	96 100
8	Wang et al. 2016 [32]	ND	ND	ND	ND	ND
9	Bashti et al. 2018 [101]	ND	ND	ND	ND	ND

NA: Data not available; ND: no ROC (Receiver operating characteristic) curves drawn.

**Table 5:** Inclusion criteria for the studies.

Parameters	Criteria
Study Design	Prospective or Retrospective Cohort or Case-Control Design with a Well-Defined Study Population
Source	Peer-reviewed journals
Language	English
Disease	Endometriosis
Sample type	Blood, serum or plasma
Technique	Microarray, qRT-PCR, NGS, ISH
Stage of disease	any
Type of endometriosis	any
Sample size	≥20

qRT-PCR: quantitative real time reverse transcription PCR; NGS: next-generation sequencing; ISH: in situ hybridisation.

## Supplementary Materials

Supplementary materials can be found at http://www.mdpi.com/1422-0067/19/2/599/s1.
