# Supplementary material for: The miRNA Mirage: How Close Are We to Finding a Non-Invasive Diagnostic Biomarker in Endometriosis? A Systematic Review
Source: Int J Mol Sci. 2018 Feb 17;19(2):599. doi: 10.3390/ijms19020599 (PMC5855821; doi:10.3390/ijms19020599)
Supplement: Supplementary file 1 [file ijms-19-00599-s001.pdf]

Table S1. Table showing literature search strategies.

Database	Search Terms
PubMed (via https://www.ncbi.nlm.nih.gov/pubmed) Search date: 14 December 2016	1. Endometriosis [mh]<br>2. endometrio * [tiab]<br>3. #1 OR #2<br>4. MicroRNAs [mh]<br>5. RNA, Antisense [mh]<br>6. RNA, Untranslated [mh]<br>7. miRNA* [tiab]<br>8. microRNA* [tiab]<br>9. micro-RNA* [tiab]<br>10. miR [tiab]<br>11. #4 OR #5 OR #6 OR #7 OR #8 OR #9 OR #10<br>12. Biomarkers [mh]<br>13. biomarker* [tiab]<br>14. "biological marker" [tiab]<br>15. "biological markers" [tiab]<br>16. "biologic marker" [tiab]<br>17. "biologic markers" [tiab]<br>18. circulat* [tiab]<br>19. "biochemical marker" [tiab]<br>20. "biochemical markers" [tiab]<br>21. "immunologic marker" [tiab]<br>22. "immunologic markers" [tiab]<br>23. "immunological marker" [tiab]<br>24. "immunological markers" [tiab]<br>25. "immune marker" [tiab]<br>26. "immune markers" [tiab]<br>27. "laboratory marker" [tiab]<br>28. "laboratory markers" [tiab]<br>29. "serum marker" [tiab]<br>30. "serum markers" [tiab]<br>31. "clinical marker" [tiab]<br>32. "clinical markers" [tiab]<br>33. #12 OR #13 OR #14 OR #15 OR #16 OR #17 OR #18 OR #19 OR #20 OR #21 OR #22 OR #23 OR #24 OR #25 OR #26 OR #27 OR #28 OR #29 OR #30 OR #31 OR #32<br>34. Serum [mh]<br>35. sera [tiab]<br>36. serum [tiab]<br>37. #34 OR #35 OR #36<br>38. Plasma [mh]<br>39. plasma [tiab]<br>40. #38 OR #39<br>41. Blood [mh]<br>42. blood [tiab]<br>43. #41 OR #42<br>44. Diagnosis [mh]<br>45. diagnos* [tiab]<br>46. detection [tiab]

	47. identif* [tiab]<br>48. discover* [tiab]<br>49. #44 OR #45 OR #46 OR #47 OR #48<br>50. Prognosis [mh]<br>51. prognos* [tiab]<br>52. predict* [tiab]<br>53. #50 OR #51 OR #52<br>54. #33 OR #37 OR #40 OR #43 OR #49 OR #53<br>55. #3 AND #11 AND #54<br>56. 1996:2016 [dp]<br>57. #55 AND #56
Embase via OVID (1974 to 2016 Week 50)<br>Search date: 14 December 2016	1. Endometriosis/<br>2. endometrio$.ti,ab.<br>3. OR/1-2<br>4. exp microRNA/<br>5. (miRNA$ OR microRNA$ OR micro-RNA$ OR miR).ti,ab.<br>6. OR/4-5<br>7. Biological marker/<br>8. (biomarker$ OR circulat$).ti,ab.<br>9. (biological adj marker$).ti,ab.<br>10. (biologic adj marker$).ti,ab.<br>11. (biochemical adj marker$).ti,ab.<br>12. (immunologic adj marker$).ti,ab.<br>13. (immunological adj marker$).ti,ab.<br>14. (immune adj marker$).ti,ab.<br>15. (laboratory adj marker$).ti,ab.<br>16. (serum adj marker$).ti,ab.<br>17. (clinical adj marker$).ti,ab.<br>18. OR/7-17<br>19. Serum/<br>20. (sera OR serum).ti,ab.<br>21. OR/19-20<br>22. Plasma/<br>23. plasma.ti,ab.<br>24. OR/22-23<br>25. Blood/<br>26. blood.ti,ab.<br>27. OR/25-26<br>28. Diagnosis/<br>29. (diagnos$ OR detection OR identif$ OR discover$).ti,ab.<br>30. OR/28-29<br>31. Prognosis/<br>32. (prognos$ OR predict$).ti,ab.<br>33. OR/31-32<br>34. 18 OR 21 OR 24 OR 27 OR 30 OR 33<br>35. 3 AND 6 AND 34<br>36. limit 35 to year = “1996–2016”

BIOSIS Citation Index<br>(searched via Web of Science)<br>Search date: 14 December 2016	1. TOPIC: (endometrio *)<br>2. TOPIC: (microRNA)<br>3. TOPIC: (miRNA *)<br>4. TOPIC: (micro-RNA *)<br>5. TOPIC: (miR)<br>6. #2 OR #3 OR #4 OR #5<br>7. TOPIC: (biomarker*)<br>8. TOPIC: ("biological marker *")<br>9. TOPIC: ("biologic marker *")<br>10. TOPIC: (circulat*)<br>11. TOPIC: ("biochemical marker *")<br>12. TOPIC: ("immunologic marker *")<br>13. TOPIC: ("immunological marker *")<br>14. TOPIC: ("immune marker *")<br>15. TOPIC: ("laboratory marker *")<br>16. TOPIC: ("serum marker *")<br>17. TOPIC: ("clinical marker *")<br>18. #7 OR #8 OR #9 OR #10 OR #11 OR #12 OR #13 OR #14 OR #15 OR #16 OR #17<br>19. TOPIC: (serum)<br>20. TOPIC: (sera)<br>21. TOPIC: (plasma)<br>22. TOPIC: (blood)<br>23. TOPIC: (diagnos *)<br>24. TOPIC: (detection)<br>25. TOPIC: (identif *)<br>26. TOPIC: (discover *)<br>27. TOPIC: (prognos *)<br>28. TOPIC: (predict *)<br>29. #19 OR #20 OR #21 OR #22 OR #23 OR #24 OR #25 OR #26 OR #27 OR #28<br>30. #18 OR #29<br>31. #1 AND #6 AND #30<br>32. YEAR PUBLISHED: (1996–2016)<br>33. #31 AND #32
